# Underwater Localization via Wideband Direction-of-Arrival Estimation Using Acoustic Arrays of Arbitrary Shape †

**DOI:** 10.3390/s20143862

**Published:** 2020-07-10

**Authors:** Elizaveta Dubrovinskaya, Veronika Kebkal, Oleksiy Kebkal, Konstantin Kebkal, Paolo Casari

**Affiliations:** 1 IMDEA Networks Institute and University Carlos III of Madrid, 28918 Madrid, Spain; 2 EvoLogics GmbH, 13355 Berlin, Germany; veronika.kebkal@evologics.de (V.K.); lesha@evologics.de (O.K.); kebkal@evologics.de (K.K.); 3 Department of Information Engineering and Computer Science, University of Trento, 38123 Povo (TN), Italy; paolo.casari@unitn.it

**Keywords:** wideband array processing, localization, direction of arrival estimation, side information, clustering, multilateration, emulation, lake experiment

## Abstract

Underwater sensing and remote telemetry tasks necessitate the accurate geo-location of sensor data series, which often requires underwater acoustic arrays. These are ensembles of hydrophones that can be jointly operated in order to, e.g., direct acoustic energy towards a given direction, or to estimate the direction of arrival of a desired signal. When the available equipment does not provide the required level of accuracy, it may be convenient to merge multiple transceivers into a larger acoustic array, in order to achieve better processing performance. In this paper, we name such a structure an “array of opportunity” to signify the often inevitable sub-optimality of the resulting array design, e.g., a distance between nearest array elements larger than half the shortest acoustic wavelength that the array would receive. The most immediate consequence is that arrays of opportunity may be affected by spatial ambiguity, and may require additional processing to avoid large errors in wideband direction of arrival (DoA) estimation, especially as opposed to narrowband processing. We consider the design of practical algorithms to achieve accurate detections, DoA estimates, and position estimates using wideband arrays of opportunity. For this purpose, we rely jointly on DoA and rough multilateration estimates to eliminate spatial ambiguities arising from the array layout. By means of emulations that realistically reproduce underwater noise and acoustic clutter, we show that our algorithm yields accurate DoA and location estimates, and in some cases it allows arrays of opportunity to outperform properly designed arrays. For example, at a signal-to-noise ratio of –20 dB, a 15-element array of opportunity achieves lower average and median localization error (27 m and 12 m, respectively) than a 30-element array with proper λ/2 element spacing (33 m and 15 m, respectively). We confirm the good accuracy of our approach via emulation results, and through a proof-of-concept lake experiment, where our algorithm applied to a 10-element array of opportunity achieves a 90th-percentile DoA estimation error of 4${}^{\circ }$ and a 90th-percentile total location error of 5 m when applied to a real 10-element array of opportunity.

## 1. Introduction

Underwater sensing and remote telemetry tasks produce the most valuable results when they can clearly geo-locate sensed data values. This is especially important when sensing the presence of acoustic signals coming, e.g., from wildlife or man-made devices: in these cases, estimating the location of the acoustic source and tracking it over time typically yields significantly valuable information, and may likely be the ultimate task of the sensing process. Common solutions for this challenging task include image- and video-based monitoring [1], LiDAR systems [2], as well as acoustic systems [3].

In underwater scenarios, accurate localization typically requires acoustic arrays. These pieces of equipment encompass multiple hydrophones or acoustic transceivers. This enables spatial filtering to increase directivity towards specific directions, and makes it possible to estimate the direction of arrival (DoA) of a signal. In several cases, underwater acoustic arrays are sizeable, and may require complex handling for deployment at sea.

When possible, the shape of an acoustic array is designed to fit the need of some application. For example, the side-scan sonar of an autonomous underwater vehicle (AUV) is usually a 2D matrix of acoustic elements [4,5] designed to cover a given aperture with a given resolution, expressed in terms of the beamwidth of the main lobe of the array’s beam patterns. Other relevant examples include linear arrays for DoA estimation [6] and three-dimensional general-purpose scanners such as tetrahedral and pyramidal arrays [7].

If arrays are modularly designed, it is typically possible to improve the spatial scanning performance by joining several arrays into a more complex layout. For example, several linear arrays could be joined into a 2D matrix, or into a 3D cylindrical configuration that makes it possible to scan a water volume and discriminate among different directions of arrival. Tesei et al. [8] showed that fusing the information from multiple arrays improves localization and ranging, even if these arrays are located far apart, and receive uncorrelated acoustic signals.

In this paper, we are interested in “acoustic arrays of opportunity.” As opposed to arrays specifically designed for a given task, acoustic arrays of opportunity are typically composed of multiple sub-arrays originally designed to work independently, where each sub-array may contain one or more hydrophones or acoustic transceivers. Sometimes, sub-arrays come as standalone units: an opportunistic array would merge and co-operate multiple such units.

The main challenge related to acoustic arrays of opportunity is that each sub-array may have a physical design or may present mounting constraints that prevent the array of opportunity from having the optimal structure for a given task. For example, we may have to ensure some minimum spacing among the sub-arrays in order to preserve connectors, or to avoid that power and data cables bend in excess of their specifications. Additionally, the sub-arrays may have pre-defined shapes, and it is typically unfeasible to reconfigure these shapes into other layouts.

There are at least two important consequences to the above constraints. First, it may be impossible to construct typical array topologies such as uniform linear arrays (ULAs), uniform rectangular arrays (URAs), or cylindrical arrays [9]. Second, the resulting layout may force a larger-than-optimal spacing among closest array elements, e.g., larger than λ/2, where λ is the wavelength corresponding to the maximum operational acoustic frequency of the array’s hydrophones. An improper spacing of the array elements causes spatial ambiguities in beamforming and DoA estimation operations. For example, multiple, equally strong lobes in the opportunistic array’s beam pattern, or equivalently, it may become impossible to distinguish among multiple equally likely DoA estimates. Additional physical characteristics of the array elements, such as a non-omnidirectional radiation pattern, may be insufficient to remove such spatial ambiguities [10]. When the purpose of the array is localization through multilateration, larger-than-λ/2 spacing may also lead to significant errors [11].

The above issues have an even larger impact when employing wideband 3D DoA estimation algorithms. Notably, most wideband algorithms [10] work with predefined array shapes, or are limited to 2D, to specific signals, or to a known number of targets [12]. In some cases, the preferred solution is to directly employ particle velocity sensors [13,14,15].

In this paper, we propose a wideband DoA estimation scheme based on the delay-sum algorithm. Our scheme that works with arbitrary array layouts, where both the 3D arrangement of the array elements and the spacing among them are potentially irregular or arbitrary. This fits well our assumption that the array opportunistically merges independent subsystems. To remove spatial ambiguity, we compute rough target location estimates via multilateration, using time difference of arrival (TDoA) measurements from the array elements. We then restrict the DoA search space to an area around the target, using multilateration estimates as side information. This rules out or at least dampens ambiguous directivity peaks. When the approach is successful, we accrue the additional advantage that peaks affected by spatial ambiguity are narrower [10], and thus yield a more accurate DoA estimate. We observe that such an approach works even if the array elements are not sufficiently spaced to achieve high resolution [8], and therefore multilateration estimates are not extremely accurate.

We evaluate our approach by running emulations and by performing a lake experiment. Emulations provide a controlled environment that simplifies acoustic propagation and detection, but recreates realistic acoustic background conditions by using real clutter noise recordings from an in-water experiment. The lake experiment, instead, involves real hardware and realistic water conditions, including small movements due to currents and waves. In both cases, our results show that our algorithm effectively estimates DoAs and 3D target locations. Using the extra freedom allowed by emulation, we also show that merging together realistic pyramidal arrays (such as those found in off-the-shelf equipment, e.g., [16]) yields better DoA estimation performance than typical cylindrical arrays having λ/2 element spacing.

In summary, our approach yields the following advantages: (i) it provides a framework to merge together smaller arrays into a larger “array of opportunity” to achieve better DoA estimation accuracy; (ii) it provides a method to rule out the ambiguity that may result from the suboptimal spacing of the array elements; (iii) it works with wideband signals and arbitrary array topologies; (iv) it yields good performance in emulated sea environments as well as in a proof-of-concept experiment. In particular, emulation results show that, at a Signal-to-Noise Ratio (SNR) of −20 dB, a 15-element array of opportunity achieves lower average and median localization error (27 m and 12 m, respectively) than a 30-element array with proper λ/2 element spacing (33 m and 15 m, respectively). In a proof-of-concept lake experiment, we additionally show that our algorithm achieves a 90th-percentile DoA estimation error of 4${}^{\circ }$ and a 90th-percentile total location error of 5 m when applied to a real 10-element array of opportunity. This realistic performance evaluation substantially extends the preliminary simulation results in our previous work [17]. Moreover, in this paper, we included additional related literature, extended the presentation and explanation of our approach with examples of intermediate steps, and provided evidence that the components of our algorithm that complement wideband DoA estimation can run in real time on an embedded platform.

The remainder of this paper is organized as follows. In Section 2, we survey relevant related work. In Section 3, we describe our DoA estimation method. We introduce materials and methods for our performance evaluation in Section 4. Section 5 and Section 6 cover the evaluation of our proposed scheme via emulation and via a lake experiment, respectively. Finally, we discuss our results in Section 7, and draw concluding remarks in Section 8.

## 2. Related Work

The engineering of array processing schemes for underwater detection and communication spans several disciplines, from sonar systems to communications and underwater target detection with either passive or active arrays [18]. Recent advances in this fields include the application of different estimation or signal processing techniques to classical beamforming algorithms, with the objective of improving their accuracy and decrease their complexity. For example, using a particle filter to estimate the DoA of an acoustic source improves the performance of Bartlett and conventional beamformers [19]. Real data from the SwellEx’96 sea experiment validate the findings of the study. Chen et al. [20] improve the performance of a blind DoA estimation algorithm from the literature [21] by exploiting partial knowledge on the structure of the signal transmitted by an acoustic source. With the aim to reconstruct the DoA of a wideband underwater signal, Tang et al. [6] use sparse signal representation and provide further methods to eliminate the aliasing originating from the over-completeness of the measurement dictionary. The authors prove the effectiveness of their algorithm using a uniform linear array to detect the breathing sounds of divers equipped with closed-circuit rebreathers.

Van Kleunen et al. [22] consider a blind node integrating a 4-element linear array for DoA estimation, and mix DoA with time of flight (ToF) information related to the signals that the blind node receives from synchronous reference nodes. On a similar vein, Guo et al. [23] employ a linear array to localize a node emitting acoustic signals, by leveraging the multipath components appearing at the receiver. Weighed subspace fitting helps avoid the explicit estimation of the DoA for each multipath arrival. Tesei et al. [8] discuss sound source localization in 3D using either one or two tetrahedral arrays deployed at different locations. Despite synchronous sampling in the two systems, their algorithm does not process the arrays jointly, as the distance between the arrays decorrelates the received signals.

Multiple works applied compressive sensing and sparse reconstruction techniques to underwater array processing. For example, Song et al. [24] use compressive beamforming to estimate the DoA of an underwater acoustic source via a forward-looking sonar, and validate the system using field experiment data. Sparse reconstruction [9] enables the estimation of the DoA of sound emitted by underwater vessels. The authors carry out an experiment using a passive towed linear array sonar that showcases the performance of their algorithm. Two-dimensional continuous compressive sensing is used in [25] to estimate the complete set of measurements of a URA starting from a sparser array. The resulting estimates are then employed to impute the missing measurements and compute the DoA of a signal impinging on the array. Coherent signal subspace processing and compressive sensing are jointly considered for wideband DoA estimation in [26]. Compared to the conventional minimum variance distortionless response (MVDR) beamformer, the proposed method yields higher resolution.

Acoustic vector sensors, also known as particle velocity sensors, provide a first estimate of the direction of arrival of an underwater signal. Owing to this, several works rely on vector sensors for DoA estimation [27,28,29]. With a focus on computationally efficient DoA estimation, Bereketli et al. [15] employ an acoustic vector sensor to estimate the DoA of an impinging signal in a shallow water scenario, where strong multipath echoes degrade the quality of DoA estimates. Vector sensors can also be arranged into arrays, and coherently processing the sensors’ signals improves the resolution of underwater DoA estimation for wideband coherent sources [30].

Wideband beamforming recently spurred significant interest in the broadband terrestrial radio communication domain [31,32,33]: as underwater acoustic systems are typically wideband, similar techiques find applications for underwater acoustic detection and communications as well. Liu and Weiss [10] extensively cover classical approaches and recent research results for wideband array processing with applications to signal enhancement and DoA estimation. Multichannel processing through diversity combining and optimal beamforming is the focus of the work in [34], which targets the reception of high-speed underwater acoustic communication signals. The authors show that beamforming enables the design of a significantly simpler receiver, which can coherently extract multipath signal energy in a sea experiment. A similar approach [35] employs mono-pulse processing to cancel incoherent multipath components that would interfere with the receiver in a reverberating shallow water acoustic communications scenario. Bayesian methods (BMs) are also applied to the estimation of the DoA of wideband linear frequency modulated (LFM) signals using a uniform linear array [36]. The authors resort to the fractional Fourier transform to extract the wanted signal from a reverberating background and improve the operating signal-to-noise ratio.

Typically, the development of signal processing algorithms for underwater acoustic arrays assumes a simple array topology, for which the steering vectors and array manifolds can be computed in close-form. Often, linear arrays are used [9,19,20,22,23,24] or rectangular arrays [25]. A study involving 3D, 4-element tetrahedral arrays is provided in [8]. Unlike the above literature, in this paper, we propose a wideband DoA estimation algorithm that works on any 3D underwater array layout. Thus, our approach encompasses imperfect array design occurrences, or the opportunistic combination of multiple arrays into a larger structure to seek better spatial performance. A key realistic assumption of our work is that array sensors may not be properly spaced at a distance of λ/2 from one another, possibly resulting in spatial ambiguity that must be compensated for.

## 3. Wideband DoA Estimation Algorithm

We now proceed to introduce and explain our wideband DoA estimation method. We start by providing the key idea behind the algorithm in Section 3.1, and continue with the details in Section 3.2.

### 3.1. Key Idea

We assume to operate an array of known topology, but whose elements are not necessarily arranged to obey the λ/2 spacing constraint. The array elements are co-located, and the array control electronics synchronously retrieve acoustic samples from all elements. The task of the array is to detect the DoA of signals with a known structure, either emitted by AUVs and other man-made equipment, or emitted by a projector co-located with the array and reflected back by the target.

Our algorithm mitigates spatial ambiguity via side information in the form of a rough location estimate derived from TDoA-based multilateration. This information helps filter the output of a wideband delay-sum DoA estimation algorithm and thus rules out most of the ambiguous DoA estimates.

### 3.2. Algorithm Description

**Figure 1:**
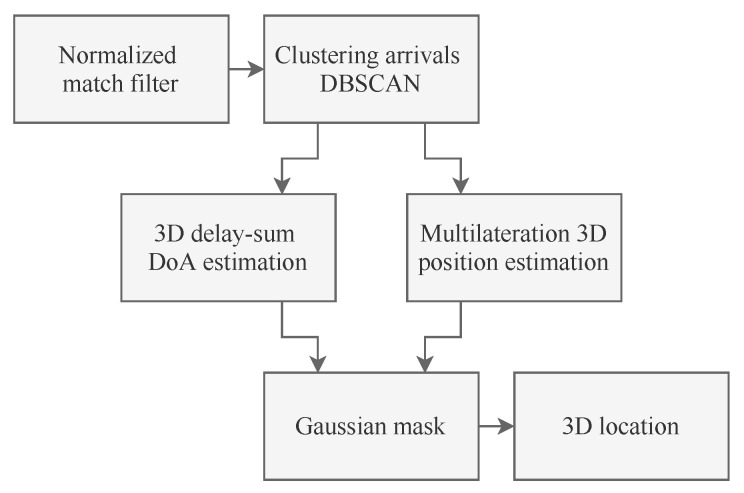
Flow diagram of the DoA estimation and localization algorithm.

Call f(t) the signal that the array seeks the DoA of. While this can be any signal, if the array is co-located with a projector and listens to reflections of the projector’s signal, a typical solution is to employ linear chirps spanning the frequency interval from $f_{\min}$ to $f_{\max}$ over a time interval of duration *T*. Such chirp would have the form $f\left(t\right)=\cos\left(2\pi \;\frac{f_{\max}-f_{\min}}{2T}\;t^{2}+f_{\min}\;t\right)$. Let $s_{n}\left(t\right)$ be the real-valued signal received by the *n*th array element. With reference to Figure 1, our scheme proceeds by first detecting f(t) within the $s_{n}\left(t\right)$ signals using a normalized matched filter (NMF) [37]. For each array element, the output of the NMF is expressed as

(1) $$R_{n}\left(\tau \right)=\frac{\int_{0}^{+\infty }f\left(t\right)\;s_{n}\left(t+\tau \right)\;d\;t}{{\left(\int_{0}^{T}f^{2}\left(t\right)\;d\;t\int_{0}^{+\infty }s_{n}^{2}\left(t\right)\;d\;t\right)}^{1/2}}\;.$$

We search relevant peaks in $R_{n}\left(\tau \right)$ via a sliding window method. In more detail, we consider a window of length *T* aligned with the beginning of $R_{n}\left(\tau \right)$, and take the highest peak in the window; then, we slide the window, take again the highest peak, and repeat the process until we cover the whole of $R_{n}\left(\tau \right)$. This filtering step eliminates secondary peaks that are never the tallest in any window. Call $P_{n}$ the set of peaks that survived filtering, where the features that fully define a peak $p\in P_{n}$ are its time of occurrence *t*, its amplitude *a* and the hydrophone *n* that detects it, i.e., $p=\left(t,a,n\right)\in P_{n}$.

We proceed by applying the DBSCAN algorithm [38] over the whole set of peaks

(2) $$P=P_{1}\cup \cdots \cup P_{n}\;.$$

DBSCAN approximates the function

(3) C=D(P)

that returns all subsets of arrivals C∈C, such that each subset *C* contains groups of detections that correspond to the same target. We choose DBSCAN because it works based on point density, which we found to be a very good indicator of target detection. In fact, when a signal from a target insonifies the array, no two NMF peaks related to this detection should be farther in time than the maximum propagation delay between any two array elements. This allows us to define the density of the NMF peaks in time across multiple channels. Moreover, DBSCAN executes very fast [39,40] on our NMF time series (typically in less than 1 ms) and the algorithm does not need prior information about the number of points that are part of a cluster, or the number of clusters in the dataset. As a result, DBSCAN is suitable for multiple target detection in scenarios with multiple targets.

We configured DBSCAN to seek arrivals detected in at least 70% of the array elements, and spaced in time no more than the maximum propagation delay between any two elements. This makes it possible to discard peaks that are not detected reliably by all elements, or that are separated by a large time delay, enough to suspect that they may correspond to different emissions from the environment, or to reflections from different targets. We remark that the threshold on the number of array elements that should detect target-related peaks configures a trade-off between the probability of missing a detection, and the probability of wrongly including a detection that pertains to a different target. In fact, some array elements may be shadowed by other sensors, cables, or structural components of the array, and therefore a cluster *C* may contain peaks from only a subset of the array elements. Additionally, if a cluster contains multiple arrivals within $t_{max}$ on the same channel, we have an option to filter the arrivals depending on the output of the normalized matched filter.

**Figure 2:**
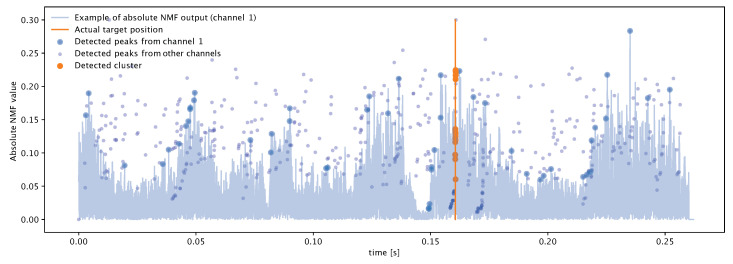
Example of successful DBSCAN clustering for peaks collected by a 10-element array. The light-blue time series is the output of the NMF for channel 1. Large blue circles represent peak detections for this NMF time series. Smaller dark-purple peaks represent peak detections from the remaining nine channels. DBSCAN correctly detects a cluster of target related arrivals around 0.16 s (vertical orange line). Data from a real lake experiment.

Using real data from a lake experiment, we show an example of clustering result for peaks collected by a synchronously-sampled 10-element array in Figure 2. The array is configured to seek linear chirp signals of duration 10 ms spanning the acoustic frequencies from 7 to 17 kHz. The light-blue time series in the background of Figure 2 is the output of the NMF for one of the ten acoustic channels, namely channel 1, depicted over time (measured in seconds). The sampling frequency is 62.5 kHz. For this channel, the peak extraction algorithm described above filters the peaks marked as a large blue circle. The same algorithm, applied to the NMF output of the remaining nine channels (whose time series are omitted for clarity), leads to the peaks marked as small, purple circles. Altogether, these peaks form set P. DBSCAN processes the peak set to detect the target. Out of all peaks, DBSCAN singles out the orange-colored ones as being likely associated with the target, due to their density and their appearance in several acoustic channels. As confirmed by the vertical orange line, these peaks correctly align with the ground truth of the target’s position, which corresponds to a time of arrival of about 0.16 s.

Once DBSCAN has detected the target, we are ready to estimate the DoA of the target’s signal. To do so, we now consider a set of elevation angles Θ and azimuth angles Φ, and scan the power received by the array along every direction identified by a pair (θ,ϕ), for θ∈Θ and ϕ∈Φ. We remark that we could set Θ=(0,π) and Φ=(−π,π) in order to cover all possible DoAs in 3D space, or rather restrict Θ and Φ to a smaller domain, in case some prior information is available.

For each cluster *C*, we measure the energy perceived by the array along different directions through a wideband delay-sum algorithm [10]. We stress that using wideband algorithms matches the possibly wideband signals detected or projected by the array. For example, the equipment in [16] operates across the bandwidth from 7 kHz to 17 kHz, which aligns well with the definition of wideband equipment. In any event, our approach also works with narrowband signals.

We implement the algorithm in the frequency domain. Specifically, we cut the output of the matched filter that covers the arrivals in cluster *C* for each hydrophone, and then apply an fast Fourier transform (FFT) to this signal chunk. We then apply a different, frequency-dependent phase shift vector to each frequency bin in order to steer the array towards the direction (θ,ϕ). Finally, we convert back to the time domain via an inverse FFT, and sum up the resulting outputs across all hydrophones.

By carrying out this operation for several 3D DoAs (θ,ϕ), we obtain a map α(θ,ϕ) of the power received over all scanning directions specified by sets Θ and Φ. As we consider opportunistic arrays where the elements may be spaced more than λ/2, the delay-sum map may be affected by ambiguities, hence it may indicate the reception of a significant amount of power from directions different than the true DoA of the target.

The key to recuse such ambiguity is to mask the above DoA map by roughly localizing the target in 3D space via a multilateration algorithm. We achieve this through TDoA measurements carried out across the array elements. Call

(4) $$u={\left[x\;y\;z\right]}^{\;T}\;\;\mathrm{and}\;\;u_{n}={\left[x_{n}\;y_{n}\;z_{n}\right]}^{\;T}$$

the Cartesian coordinates of the target and those of hydrophone *n*, respectively. Furthermore, call $t_{0}$ the time of occurrence of the earliest peak in cluster *C*. Without loss of generality, assign index 0 to the hydrophone that receives this arrival. Finally, call *c* the sound speed near the array. We assume that such speed is known, typically via local equipment such as a sound velocity profiler or a CTD sensor.

For each peak p=(t,a,n)∈C, the corresponding multilateration equation is

(5) $$x\cdot X_{n}+y\cdot Y_{n}+z\cdot Z_{n}+D_{n}=0\;,$$

where

(6) $$X_{n}=\frac{2x_{n}}{c\;t}-\frac{2x_{0}}{c\;t_{0}}\;,\;Y_{n}=\frac{2y_{n}}{c\;t}-\frac{2y_{0}}{c\;t_{0}}\;,\;Z_{n}=\frac{2z_{n}}{c\;t}-\frac{2z_{0}}{c\;t_{0}}\;,$$

and

(7) $$D_{n}=c\;\left(t-t_{0}\right)-\frac{x_{n}^{2}+y_{n}^{2}+z_{n}^{2}}{c\;t}+\frac{x_{0}^{2}+y_{0}^{2}+z_{0}^{2}}{c\;t_{0}}\;.$$

Collecting one equation such as (5) for every peak in cluster *C* results in an over-determined system of equations, which we solve through Moore–Penrose’s pseudo-inverse. The result is a rough estimate of the target location $u^{★}={\left[x^{★}\;y^{★}\;z^{★}\right]}^{\;T}$, which we convert to polar coordinates to yield the estimated location of the target, namely

(8) $$\bar{u}={\left[\bar{r}\;\bar{\theta }\;\bar{\phi }\right]}^{\;T}\;.$$

We exploit the above estimate to define a masking function having the shape of a truncated bi-variate Gaussian kernel

(9) $$m\left(\theta ,\phi \right)=\min\left\{1;\;\frac{1}{2\pi \sigma_{\theta }\sigma_{\phi }}\;e^{-\frac{{\left(\theta -\bar{\theta }\right)}^{2}}{2\sigma_{\theta }^{2}}}e^{-\frac{{\left(\phi -\bar{\phi }\right)}^{2}}{2\sigma_{\phi }^{2}}}\right\}\;,$$

where $\sigma_{\theta }=\pi /8$ and $\sigma_{\phi }=\pi /4$. Using m(θ,ϕ), we mask the output of the wideband delay-sum beamformer in order to mitigate (and typically fully remove) ambiguities. Finally, we set the estimated DoA for the received signal as

(10) $$\hat{\theta },\hat{\phi }=\underset{\theta ,\phi }{arg max}\alpha \left(\theta ,\phi \right)\;m\left(\theta ,\phi \right)\;.$$

**Figure 3:**
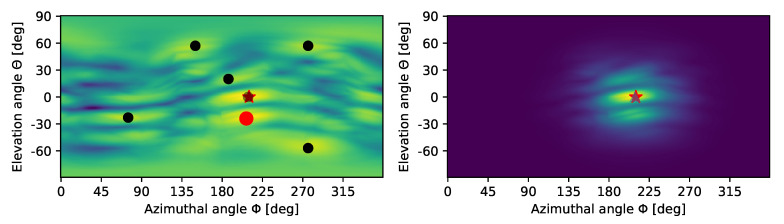
Intensity map at the output of the wideband delay-sum beamformer without (**left**) and with (**right**) TDoA multilateration-based masking. The latter mitigates the ambiguity and makes it possible to correctly estimate the location of the target (red star), while ruling out the strongest peak (red dot) which would correspond to a wrong target location. Yellow hues denote a stronger signal.

Figure 3 (left panel) provides an example of the delay-sum output. Specifically, for a number of 3D DoAs characterized by a pair of angles ϕ (azimuthal angle, *x*-axis) and θ (elevation angle, *y*-axis), we steer the array towards each DoA using wideband delay-sum beamforming, and depict the normalized amount of power at the output of the array. Yellow hues correspond to a strong signal, green hues to a signal of intermediate power, and blue hues to a weak or absent signal. Notably, there exist several local maxima (black dots surrounded by yellow-green hues), which make the decision ambiguous. In fact, measurement noise in conjunction with ambiguity would lead to a wrong estimate (red circle) of the target’s actual DoA (red star).

In the right panel of Figure 3, instead, we apply the multilateration-based mask. As explained above, we employ TDoA information extracted from the peaks that DBSCAN recognizes as being part of the same target detection (formally, cluster *C* above). These peaks come from different acoustic elements within the array: computing the TDoA values for these peaks makes it possible to roughly localize the target via multilateration, and to construct the mask in Equation (9). This filters out most of the ambiguity and points to the correct DoA (red star). We note that some local maxima still remain even after applying the mask (corresponding to the green hues above and below the starred peak). However, these peaks are now sufficiently mitigated, and do not impede a correct DoA estimation.

As a final step, we fuse the estimated DoA with ranging information and pass it on as a valid location only if the position of the target remains within the boundaries of the water column.

## 4. Materials and Methods

In this section, we summarize the methods and materials used for the performance evaluation of our proposed DoA estimation algorithm. After a short account of common assumptions in the two evaluation setups, we present our emulation framework in Section 4.2 and our experiment framework in Section 4.3. Section 5 and Section 6 follow up with the results of the corresponding performance evaluations.

### 4.1. Common Setup and Parameter Configurations

In the following, we assume that our array of opportunity seeks linear chirp signals in order to localize a nearby target. Each chirp has duration T=10 ms and spans the acoustic band from $f_{\min}=7$ kHz to $f_{\max}=17$ kHz. We also assume that the array can synchronously sample its elements, and can store the corresponding acoustic samples for immediate or offline processing.

### 4.2. Emulation

The key idea of the emulation is to employ measurements of noise and acoustic clutter from a lake experiment, in order to achieve a more realistic representation of the signal received by the array elements.

Specifically, we consider an experiment performed in the Werbellin freshwater lake, located 60 km north of Berlin, Germany. During this experiment, we acquired several underwater acoustic recordings containing environmental noise and clutter. We subdivide these recordings in chunks of 16 ms (equal to 1000 samples at a sampling frequency of 62.5 kHz) and normalize each chunk so that the standard deviation of the noise is equal to 1 throughout all chunks. Finally, we create a Monte Carlo set of emulated noise recordings, where in each recording we randomly shuffle the order of the 16-ms noise chunks.

Our emulation framework consists of a software written in Python. Here, we assume that the array of opportunity is located at a depth of 10 m within an isovelocity water body with flat surface and bottom having maximum depth of 100 m. (The lake experiment in Section 6 serves to test our algorithm in realistic propagation conditions, with a stratified medium and non-flat bottom.) For the emulation scenario, we assume that the array insonifies the underwater environment by transmitting the chirp signal, and listens to reflections from the environment. We thus emulate a received signal by propagating the chirp to the target, and back to the array. In particular, we shift the phase of the chirp as a function of the location of the source and of the position of each acoustic array element. We then scale a noise sequence from the Monte Carlo set to yield a desired SNR level, and superimpose the received signal to the noise. Finally, we apply our DoA estimation algorithm to the signal. We repeat the experiment for 270 different locations of the target, chosen to represent all array lookout directions. For each location, we repeat the estimation for ten different underwater noise realizations.

**Figure 4:**
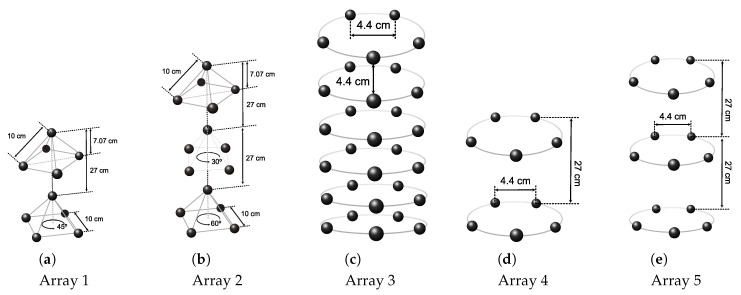
Array topologies considered in this paper.

We consider five different array layouts, as illustrated in Figure 4:

- Array 1 is composed of two 5-element pyramidal arrays having a base side length of 10 cm and an height of 7.07 cm. The sub-arrays are stacked at a distance of 27 cm, and the bottom one is rotated by 45${}^{\circ }$. This is typical in the case in which each 5-element pyramidal array actually comes as a separate unit, whose connector mounting and cable bending constraints prevents placing the units closer than a given maximum distance;
- Array 2 is similar to array 1 but is composed of three pyramidal arrays stacked at a distance of 27 cm. In this case, the second array is rotated by 30${}^{\circ }$ and the third by 60${}^{\circ }$;
- Array 3 is a cylindrical array composed of 6 circular sub-arrays of 5 elements each (the same number of elements as in the pyramidal arrays of Array 1 and Array 2). The distance between closest elements along the same ring and across different rings is 4.4 cm;
- Array 4 is composed of two circular sub-arrays of radius 3.5 cm, placed at a distance of 27 cm from each other. Each sub-array embeds 5 elements. The elements are equally spaced along the ring and closest elements are 4.4 cm apart;
- Array 5 is similar to array 4 but is composed of three rather than two rings.

We chose the topologies of acoustic arrays 1 and 2 above as they resemble closely the arrays of opportunity attached to the underwater fauna detection platform described in Section 4.3.1, and used for the lake experiment in Section 6. Array 3 is a typical cylindrical array. Its shape enables spatial scanning along both the azimuthal plane and the elevation plane; moreover, the spacing between closest array elements is less than or equal to λ/2 up to a frequency of 17 kHz. Finally, arrays 4 and 5 are also cylindrical arrays, but the distance between subsequent circular 5-element sub-arrays is 27 cm, in order to emulate the performance of Array 3 in case it had the same number of elements and mounting constraints as arrays 1 and 2.

We remark that assuming a sound speed of 1500 m/s, a distance of 4.4 cm corresponds to λ/2 spacing up to a frequency of ≈17 kHz. Because array 3 is designed with proper λ/2 spacing throughout the whole bandwidth of the chirp signal, we do not apply the m(θ,ϕ) mask to the wideband delay-sum output in this case.

**Figure 5:**
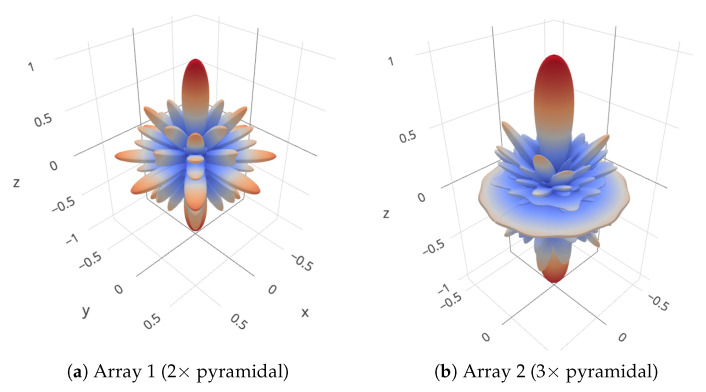
Examples of ambiguity in the directivity pattern for arrays 1 and 2 at frequency $f=f_{\max}$, once steered towards the θ=0 direction.

As an example of the ambiguity originating from the opportunistic array design and the suboptimal element spacing that ensues, in Figure 5, we show the 3D directivity pattern at frequency $f_{\max}$ for Arrays 1 and 2, as steered towards the θ=0 direction. In both cases, we observe several secondary peaks almost as strong as the tallest peak. These peaks originate primarily from the 27-cm spacing between subsequent pyramidal arrays, as such distance is about 7 times the appropriate λ/2 spacing of 4.4 cm. In Section 5 and Section 6, we show that our algorithms still obtain meaningful location estimates even when using the equipment in the above configuration.

### 4.3. Lake Experiment

#### 4.3.1. Equipment and Software

**Figure 6:**
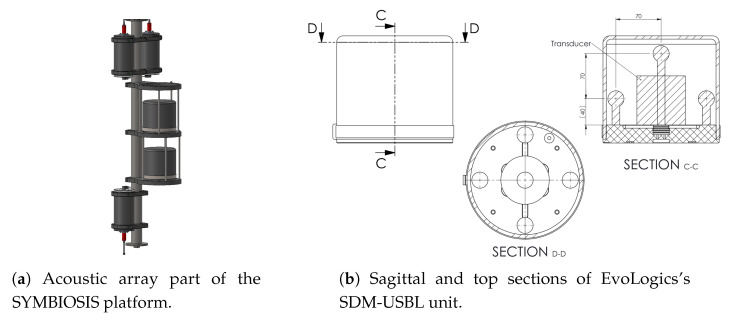
(**a**) rendering of part of the upper portion of SYMBIOSIS platform, showing the acoustic array of opportunity employed in our experiment (two cylindrical SDM-USBL units, facing right); (**b**) internal configuration of an SDM-USBL unit. Each sphere denotes a receiving acoustic element (5 in total, arranged into a pentahedral, square-base pyramid). The unit includes a transducer (the large cylindrical element in the sagittal C-C section), not used in our setting.

The acoustic array we deployed for this experiment is a part of the opto-acoustic system built in the scope of SYMBIOSIS project [41] for the non-invasive monitoring of coastal and deep waters. The purpose of the platform is to detect, localize, and monitor fish stock from different target pelagic fish species, using a chain of acoustic and optical detection systems and algorithms. Figure 6a shows a rendering of the acoustic components in the upper portion of the SYMBIOSIS instrumentation. The top and bottom cylinders host control hardware and batteries to operate the platform, whereas the two cylindrical pieces of equipment facing right constitute the acoustic array considered in our experiment. Each cylinder contains a software-defined ultra-short baseline (USBL) with modem capabilities (SDM-USBL). Figure 6b shows the internal geometry of the unit via one sagittal and one longitudinal section. Each SDM-USBL consists of a modem transducer (at the geometric center of the USBL grid) and five receive hydrophones that surround it. The hydrophones form a pentahedral, square-base pyramid having side length of 10 cm, with one hydrophone per vertex. A set of commands enables the control of each SDM-USBL. Relevant capabilities include: (i) storing a user-defined signal with a duration of 1024 samples (two bytes per sample) with a sampling frequency of 62.5 kHz; (ii) setting the unit into a listening mode, where each hydrophone digitizes its received sound signal synchronously and stores the corresponding samples into a buffer holding 51,200 samples per channel; the sampling rate is user-defined, although the default rate of 62.5 kHz perfectly suits our deployment; (iii) reading acoustic data from the buffers; and (iv) transmitting the stored signal through the central transducer.

Each SDM-USBL can operate in an “active” or “passive” mode. By triggering the “active” mode, the SDM-USBL sends the stored user-defined signal and then stores 51,200 samples per acoustic channel; in the “passive” mode, instead, the unit does not transmit any signal, but rather starts recording immediately. A sync-in signal allows us to trigger the two units at the same time, thus sampling synchronously from both of them (10 channels in total).

While each stand-alone USBL would natively work as a fully capable localization device, the SDM-USBL option and the external sync-in signals implemented by EvoLogics in the context of SYMBIOSIS disable the USBL firmware, and rather make it possible to collect acoustic samples synchronously from all channels. Therefore, each unit can double as an acoustic array, and the synchronous use of multiple SDM-USBL units effectively results in an array of opportunity, whose arrangement makes it equivalent to array 1 in Section 5.

The setup of the SYMBIOSIS platform includes an NVidia Jetson TX2 board, mainly used to control the optical components and run image recognition algorithms, which are outside the scope of this paper. In our setting, we performed all signal processing steps offline. Through one of the Jetson’s general-purpose input–output (GPIO) pins, we issued the sync-in signal periodically in order to synchronously start recording from all of the 10 array hydrophones, while at the same time transmitting from the active target. This resulted in one acoustic time series of 0.7 s per acoustic channel. We measured the accuracy of the sync-in signal to be on the order of 200 ns, which is sufficiently accurate, given the sampling frequency of 62.5 kHz.

**Figure 7:**
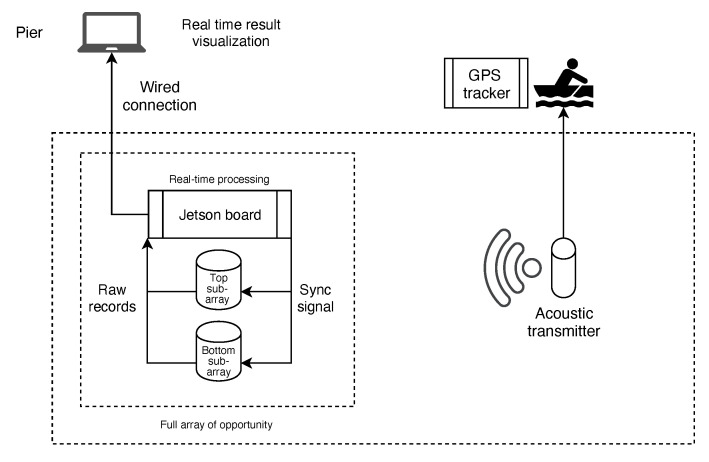
Conceptual organization of the experiment.

**Figure 8:**
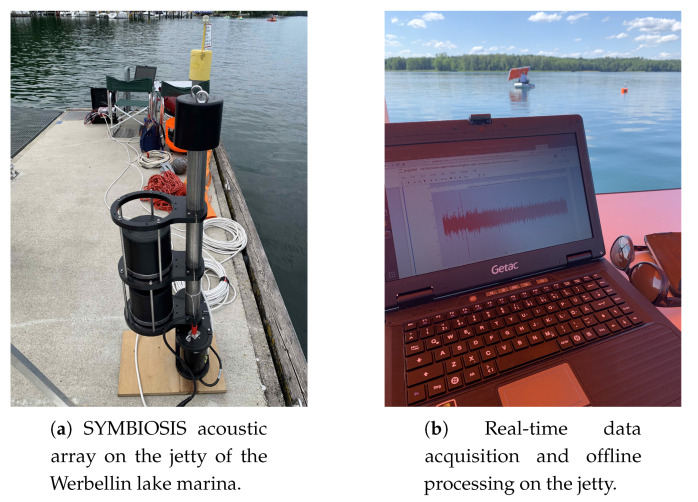
Photos of the deployment: (**a**) acoustic array of the SYMBIOSIS platform on the jetty, before deployment; (**b**) ongoing experiment, showing a snapshot of a captured signal on the laptop’s screen.

With reference to the scheme in Figure 7, we control the equipment from the laptop ashore (see also Figure 8b) using custom Python software. Once we issue the sync-in signal, the Jetson board collects data in real time, and uploads them to the laptop ashore. The software then processes the acoustic time series through the procedure of Section 3 to obtain DoA estimates.

#### 4.3.2. Experiment Setup

We performed our experiment on 13 June 2019. The weather remained mostly sunny throughout the day, with little wind. The water temperature ranged from 19.4 ${}^{\circ }$C at the bottom, up to 21.5 ${}^{\circ }$C near the lake’s surface.

**Figure 9:**
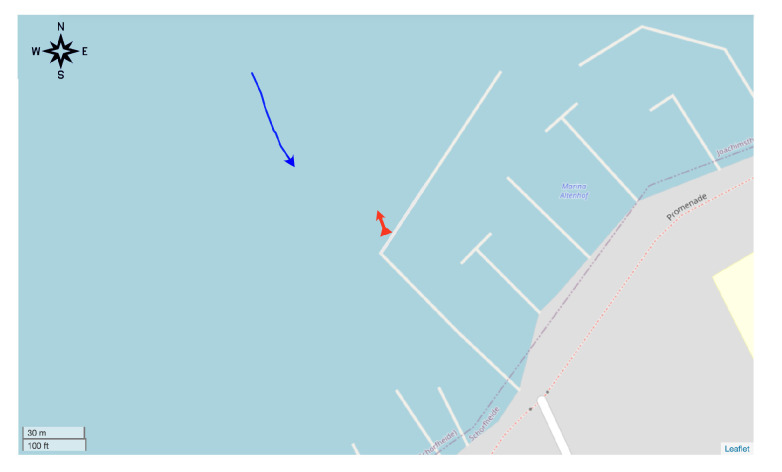
Geographical map of the experiment site near the Werbellin lake marina, Germany. The red arrow on the jetty represents the location and the reference (i.e., 0${}^{\circ }$) direction of the acoustic array; the blue line and arrow represent the trajectory and movement direction of the target throughout the experiment.

Figure 9 sketches the deployment configuration: we lowered the SYMBIOSIS unit in the water near the jetty of the Werbellin lake marina (Figure 8a), and placed it on the lake bottom at a depth of 7.5 m, so that the two units that constitute the array of opportunity remained submerged at a depth of 6.7 m and 7.1 m, respectively. The red arrow in Figure 9 denotes the location of the equipment and the reference (i.e., 0${}^{\circ }$) direction of the acoustic array. Additionally, we deployed a small motorboat carrying an active target, namely a software-defined modem emitting linear chirp signals with a duration of 10 ms in the 7–17 kHz band. The target transmitted one such signal every 2 s. The objective of this setup is to mimic the behavior of autonomous underwater vehicles that issue heartbeat signals at fixed intervals, in order to signal their presence and operational status. The task of the array is thus to estimate and track the bearing of the chirp source. In our experiment, the depth of the active target is assumed to be known, and is fixed to approximately 8 m. This is coherent with, e.g., the detection of underwater vehicles or similar equipment, which typically embed accurate depth sensors, and can communicate the corresponding data. An operator paddled the boat with the target towards the acoustic array and slightly to the side throughout the experiment, as also seen from the ground-truth trajectory of the target (the solid blue line in Figure 9), and the reference orientation of the array (the red arrow in the same figure).

## 5. Emulation-Based Performance Evaluation

We now evaluate the performance of our algorithm in an emulated underwater environment, according to the emulation setup described in Section 4.2.

**Figure 10:**
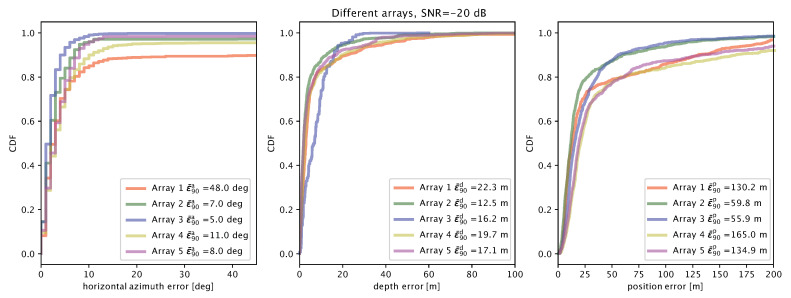
Localization error results for arrays 1 to 5, at an SNR of −20 dB: azimuthal angle error (**left**); depth error (**center**); and total location error (**right**).

Considering a target located at a distance of 100 m and a receive SNR of −20 dB, Figure 10 shows the cumulative distribution function (CDF) of the azimuthal angle estimation error (left panel), of the depth estimation error (central panel) and of the global location error (right panel) for the five array layouts introduced above. The most interesting result is that array 2 (opportunistic 3 × 5-element pyramidal arrays) achieves at least the same global location accuracy as array 3 (6 × 5-element rings with λ/2 spacing at 17 kHz), and rather shows better median and 80th percentile results, even if array 3 has 30 elements, and array 2 has only 15. We can conclude that our technique effectively coalesces the receptions of all elements in an array of opportunity, and yields good location performance by filtering out spatial ambiguities.

In more detail, we observe that array 3 achieves the best azimuth estimates as expected: e.g., it outperforms arrays 1 and 2, owing to the larger number of elements placed along the azimuthal plane. It also yields a lower maximum depth estimation error, but the rest of the CDF is better for the other arrays, which are vertically longer and favor the multilateration approach. Array 2 also achieves a marginally but noticeably lower azimuth error than array 1 because its 5-element pyramidal arrays are rotated by $30^{\circ }$ and $60^{\circ }$, respectively, which yields a better discrimination capability over the azimuthal plane.

The results suggest that arrays with elements at different heights typically perform better than shorter arrays. For example, arrays 2 and 5 (having elements at 5 and 4 different heights, respectively) outperform arrays 1 (four vertical elements) and 4 (three vertical elements). Array 2 also achieves the best 90th-percentile error (12.5 m). The good azimuth and depth estimation accuracy of array 2 makes it a very good replacement of array 3. Arrays 1, 3, and 4 show a larger maximum error, but the performance up to the third quartile is comparable with that of array 3: this is remarkable, considering that arrays 1, 3, and 4 have only 10 or 15 elements.

**Figure 11:**
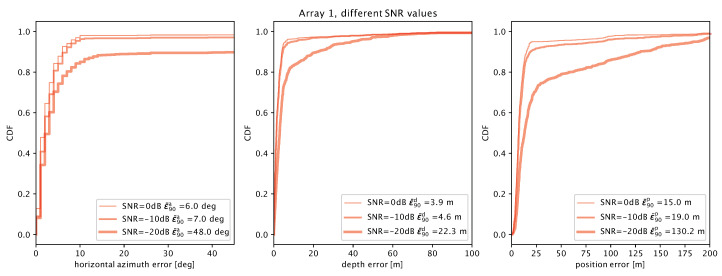
Localization error results for array 1 at different SNRs of 0, −10 and −20 dB: azimuthal angle error (**left**); depth error (**center**); and total location error (**right**).

**Figure 12:**
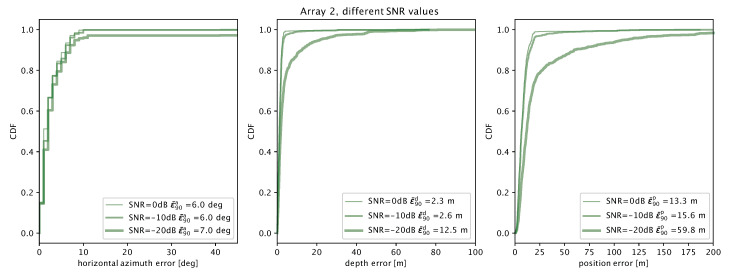
Localization error results for array 2 at different SNRs of 0, −10 and −20 dB: azimuthal angle error (**left**); depth error (**center**); and total location error (**right**).

We analyze the performance of arrays 1 and 2 in more depth in Figure 11 and Figure 12, respectively. Here, we consider three different SNR levels of 0, −10 and −20 dB. For array 1, we observe that the accuracy decreases significantly only for the SNR level of −20 dB, for which the azimuthal angle and the depth estimation errors concur to yield a large 90th-percentile error of about 130 m.

The case is different for array 2: with respect to array 1, the better multilateration performance yields comparably accurate azimuthal angle estimates for all SNR values and slightly better depth estimates even at an SNR of −20 dB. Altogether, these improvements drive the global location error to a 90th percentile of about 60 m, which is significantly lower than array 1’s.

From the above results, we conclude that the triple pyramidal structure of array 2 yields the best trade-off between azimuthal and elevation angle estimation capabilities among the tested array layouts, provided that side information is available to correct the ambiguity arising from the spacing larger than λ/2 (such as our TDoA-based mask). More broadly, we also conclude that our algorithm is a promising solution to achieve satisfactory array performance when multiple smaller sub-arrays are opportunistically combined into a larger array.

## 6. Lake Experiment Results

In this section, we present the results of the lake experiment whose configuration and setup are described in Section 4.3.

**Figure 13:**
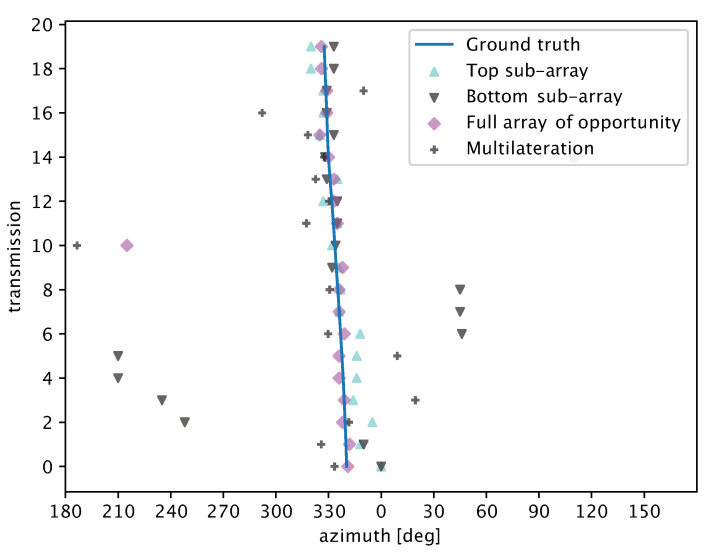
Results of the target localization experiment in the Werbellin lake using the SYMBIOSIS array of opportunity (cf. Section 4.3.1). While moving, the active target transmits every 2 s for 20 times. Each marker represents the azimuthal angle of arrival estimate (*x*-axis coordinate) for each transmission and for the corresponding array (light blue triangles: top sub-array; dark-grey triangles: bottom sub-array; purple diamonds: full array of opportunity). Grey “+” markers show the azimuth estimate yielded by multilateration. Our algorithm enables the opportunistic use of two pyramidal arrays, and makes it possible to improve the azimuth estimation accuracy with respect to using a single sub-array or multilateration per se.

We start with Figure 13, which shows azimuthal angle estimates for different array processing techniques applied to the acoustic array of opportunity that is part of the SYMBIOSIS platform. We recall that we assume the depth of the target to be fixed and equal to 8 m throughout the experiment. We apply our algorithm to different parts of the array, and specifically: (i) only to the top sub-array (light blue triangles facing up), (ii) only to the bottom sub-array (dark-grey triangles facing down), and (iii) to the full array of opportunity (purple diamonds). Grey “+” markers show the angle estimates yielded by multilateration. Throughout the experiment, the target transmits 20 signals, numbered from 0 to 19 in Figure 13. Each marker thus indicates the azimuthal angle of arrival estimate (*x*-axis coordinate) for each transmission and for the corresponding array. We also depict a solid blue line to denote the ground truth of the target’s angle of arrival as inferred from the GPS tracker’s readings (cf. Figure 7).

We observe that our method estimates the angle of arrival from the target very well. On the one hand, it avoids the sometimes largely erroneous estimates that would be computed by using only either of the two pyramidal sub-arrays (e.g., the erroneous estimates around 50 and 210–240 degrees). On the other hand, our technique fuses information from the two sub-arrays, achieving a more accurate estimate, even when each sub-array would already be satisfactorily accurate. For example, the latter is the case of the sets of readings corresponding to transmissions 0, 1, 18, and 19 in Figure 13. Notably, resorting only to multilateration would not yield accurate results, as observed from the several markers located off the ground truth line. Still, multilateration is a good source of side information for ambiguous peak removal, and helps discriminate among different equivalent peaks even when the estimate is slightly off, as for transmissions 3, 5, 16, and 17. Only for transmission 10 does the inaccurate multilateration estimate offset the DoA estimate.

**Figure 14:**
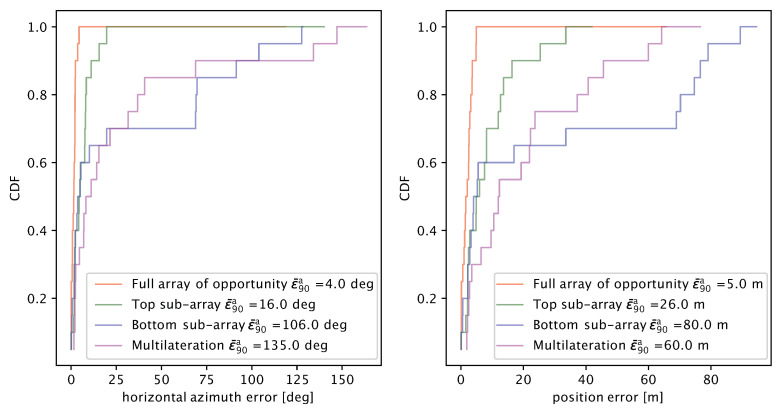
CDF of the azimuthal angle estimation error (**left**) and of the total location error (**right**) achieved in the lake experiment, showing the performance of our method as applied to different portions of the array of opportunity, as well as multilateration.

We summarize the statistics of the experimental results in Figure 14, which shows the CDF of the azimuth estimation error (left panel) and of the total location error (right panel) for all configurations considered in Figure 13. Operating the full array of opportunity with 10 elements consistently yields the most accurate results. In particular, the 90th percentile of the azimuth error (also shown in the legend for clarity) is only 4${}^{\circ }$, as opposed to 16${}^{\circ }$ when operating only the top array, 106${}^{\circ }$ when operating only the bottom array, and 135${}^{\circ }$ when resorting to pure multilateration in order to localize the target. The only large outlier for the full array of opportunity corresponds to transmission 10’s wrong multilateration estimate, which induces a wrong masking of the delay-sum estimator’s output and offsets the angle estimate.

As expected, accurate azimuthal angle estimates translate into more accurate total location error estimates. In our experiment, we achieved a 90th percentile of the location error of 5 m using the full array of opportunity. This value is 5 to 16 times less than the error yielded by operating only the top and bottom sub-arrays, respectively, and 12 times less than the error yielded by multilateration.

## 7. Summary of Results and Discussion

The results presented in Section 5 and Section 6 show that our solution is a viable underwater observation and telemetry approach. Our key proposition revolves around the concept of acoustic array of opportunity: our algorithm is designed to exploit multiple acoustic sub-arrays or sets of acoustic receivers operating together, and rules out spatial ambiguity issues by leveraging side information from rough location estimates.

The most important implication is that an array of opportunity achieves similar performance as other standard arrays having a larger number of receiving elements and a properly designed topology. This is shown in Section 5, where a 15-element array of opportunity is shown to outperform or yield equivalent results as a 30-element cylindrical array. Besides this advantage, our technique may obtain extra value from underwater reception equipment that would otherwise be impossible to merge into an array, yielding economical benefits.

Our experiment results also show that relying only on multilateration would not yield results as good as our algorithm (e.g., our scheme yields a 90th-percentile location error of 5 m, whereas multilateration yields 60 m). Our approach, therefore, can harvest the value of multilateration as side information to improve the accuracy of wideband DoA estimation.

As a final remark, our scheme embeds a wideband delay-sum DoA estimator, which lower-bounds our complexity and running time. However, all additional components, such as DBSCAN and the multilateration step, complete their execution in a negligibly small time. For example, the Julia [42] implementations of the bandpass filter, normalized matched filter, clustering, and multilateration steps complete in approximately 3.7 ms, 10.8 ms, 0.6 ms, and 0.05 ms, respectively, when run on the CPU of the Jetson TX-2 board employed in our experiments. We note that the Jetson’s CPU is much slower than the CPU of the laptop we used to process acoustic receptions.

## 8. Conclusions

In this paper, we presented a wideband DoA estimation algorithm for arrays of opportunity that coalesce smaller sub-arrays into a larger array, possibly not respecting optimal spacing constraints. We proposed to solve the spatial ambiguity issues that affect such arrays by augmenting a delay-sum DoA estimation algorithm with side information from multilateration.

Our results show that the proposed scheme yields very 3D DoA estimation error, and therefore good 3D localization results. We test our algorithm both emulating real signal reception with the help of actual clutter and noise recordings and in a lake experiment using real underwater arrays in a realistic setting. In both cases, our results show that our algorithm is robust and achieves consistently good estimation performance, often requiring a lower number of elements than typical array topologies with proper λ/2 spacing.

Future work along the lines of this paper includes a real-time implementation and test of our algorithm on embedded computers. We also plan sea experiments with different types of targets as well as with larger arrays.
